# Possible Involvement of Smad Signaling Pathways in Induction of Odontoblastic Properties in KN-3 Cells by Bone Morphogenetic Protein-2: A Growth Factor to Induce Dentin Regeneration

**DOI:** 10.1155/2012/258469

**Published:** 2012-02-28

**Authors:** Ayako Washio, Chiaki Kitamura, Takahiko Morotomi, Masamichi Terashita, Tatsuji Nishihara

**Affiliations:** ^1^Division of Pulp Biology, Operative Dentistry, and Endodontics, Department of Cariology and Periodontology, Kyushu Dental College, Kitakyushu, Japan; ^2^Section of Operative Dentistry and Endodontology, Department of Odontology, Fukuoka Dental College, Fukuoka, Japan; ^3^Division of Comprehensive Dentistry, Department of Clinical Communication and Practice, Kyushu Dental College, Kitakyushu, Japan; ^4^Division of Infections and Molecular Biology, Department of Health Promotion, Kyushu Dental College, Kitakyushu, Japan

## Abstract

We examined the effects of bone morphogenetic protein-2 (BMP-2) on growth, differentiation, and intracellular signaling pathways of odontoblast-like cells, KN-3 cells, to clarify molecular mechanisms of odontoblast differentiation during pulp regeneration process. After treatment with BMP-2, the cell morphology, growth, alkaline phosphatase (ALP) activity, and the activation and expression of BMP-induced intracellular signaling molecules, such as Smad1/5/8 and Smad6/7, as well as activities of dentin sialoprotein (DSP) and dentin matrix protein 1 (DMP1), were examined. BMP-2 had no effects on the morphology, growth, or ALP activity of KN-3 cells, whereas it induced the phosphorylation of Smad1/5/8 and expression of Smad6/7. BMP-2 also induced the expressions of DSP and DMP-1. Our results suggest that KN-3 cells may express an odontoblastic phenotype with the addition of BMP-2 through the activation of Smad signaling pathways.

## 1. Introduction

When dental pulp is exposed to external stimuli such as bacterial infection and following restorative procedures, a wound healing process is induced, and surviving odontoblasts and odontoblast-like cells differentiated from progenitor or stem cells form reactionary and reparative dentin to block further external stimuli [1–3]. During the regeneration process of dental pulp, similar mechanisms to form new dentin are also induced. Previously, it was demonstrated that controlled release of fibroblast growth factor-2 (FGF-2) from gelatin hydrogel beads induced regeneration of dentin and pulp tissue on amputated dental pulp [4, 5]. Furthermore, applications of bone morphogenetic proteins-2 and -4 (BMP-2,-4) along with dentin powder [6] and BMP-2 along with dental pulp stem cells [7] induced regeneration of dentin on amputated dental pulp. Several studies have focused on clarification of a common mechanism existing in wound healing and regeneration of dental pulp in order to overcome the limitations of present methods to preserve vital dental pulp and develop effective pulp regeneration therapies; however, the molecular mechanisms of odontoblastic cells activated by BMP-2 are not fully understood.

 BMPs are known to have diverse biological functions during embryonic development [8, 9] and osteogenesis [10, 11]. Notably, the regulation mechanisms of Runx2/Smad and other cascades controlled by BMP-2 during osteoblast and odontoblast differentiation from mesenchymal stem cells have been extensively studied [12–17]. In addition, the combination of BMP-2 with a collagen sponge was recently approved by the US Food and Drug Administration for clinical use such as oral maxillofacial surgery [18], while further studies have focused on development of an effective method based on available clinical data as well as to understanding the effects of BMP-2.

 We previously established a proliferating pulp progenitor cell line (KN-3 cells) from dental papilla cells of rat incisors [19]. KN-3 cells have high levels of alkaline phosphatase (ALP) activity and expression of Runx2 and dentin sialophosphoprotein (DSPP). The addition of the medium including ascorbic acid and *β*-glycerophosphate induced the formation of extracellular mineralized nodules, which was suppressed by bacterial lipopolysaccharides. These findings indicated that KN-3 cells exhibited typical odontoblastic properties [19]. To understand molecular mechanisms of the differentiation of KN-3 cells into functional odontoblast-like cells, we examined the effects of BMP-2 on the cell growth, differentiation, and the involvement of Smads signaling pathways in the responses of KN-3 cells on BMP-2.

## 2. Materials and Methods

### 2.1. Cell Culture

 A rat clonal dental pulp cell line with odontoblastic properties (KN-3) was maintained in alpha-modification of Eagle's medium (*α*-MEM) (Invitrogen Life Technology, Carlsbad, CA) containing 10% heat-inactivated fetal calf serum (FCS), 100 *μ*g/mL of streptomycin, and 100 U/mL of penicillin in a humidified atmosphere of 5% CO_2_ at 37°C [19].

### 2.2. Morphological Analysis

 KN-3 cells (3 × 10^4^/well) were subcultured in 6-well plates for 24 hours in *α*-MEM containing 10% FCS, 100 *μ*g/mL of streptomycin, and 100 U/mL of penicillin, then treated with BMP-2 (100 ng/mL) in *α*-MEM containing 5% FCS, 100 *μ*g/mL of streptomycin, and 100 U/mL of penicillin in a humidified atmosphere of 5% CO_2_ at 37°C. After 72 hours, the cells were observed by phase-contrast microscopy.

### 2.3. Cell Viability Assay

 The cell proliferation reagent WST-1 was used for quantitative determination of cellular proliferation (Dojindo, Kumamoto, Japan). KN-3 cells (2 × 10^4^/well) were subcultured in 96-well plates for 3 hours in *α*-MEM containing 10% FCS, 100 *μ*g/mL of streptomycin, and 100 U/mL of penicillin, then treated with BMP-2 (0–100 ng/mL) in *α*-MEM containing 5% FCS, 100 *μ*g/mL of streptomycin, and 100 U/mL of penicillin in a humidified atmosphere of 5% CO_2_ at 37°C for 48 hours. WST-1 and 1-Methoxy PMS (10 *μ*L/well) were added and incubation was performed for 2–4 hours, after which the viability of KN-3 cells was analyzed by measuring optical density with a microplate reader (Model 680; Bio-Rad laboratories, Inc., Tokyo, Japan) using a test wavelength of 450 nm.

### 2.4. Alkaline Phosphatase Activity

 KN-3 cells (1 × 10^4^/well) were subcultured in 96-well plates for 24 hours in *α*-MEM containing 10% FCS, 100 *μ*g/mL of streptomycin, and 100 U/mL of penicillin, and then treated with BMP-2 (100 ng/mL) in *α*-MEM containing 0.1% FCS, 100 *μ*g/mL of streptomycin, and 100 U/mL of penicillin in a humidified atmosphere of 5% CO_2_ at 37°C. After 1, 3, 5, and 7 days, the cells were solubilized with 200 *μ*L of Hank's salt solution containing 0.2% Nonidet P-40 (Pierce Biotechnology, Rockford, IL) for 10 minutes at 37°C. ALP activity of the lysate was measured using *p*-nitrophenylphosphate with the Lowry method. After 30 minutes of incubation at 37°C, the absorbance of *p*-nitrophenylphosphate at 405 nm was determined by using a microplate reader, and the specific activity of ALP (*μ*g/*μ*g of protein/30 minutes) was calculated. Protein contents were measured with a DC protein assay kit (Bio-Rad Lab, Hercules, CA).

### 2.5. Western Blot Analysis

 KN-3 cells treated with BMP-2 were washed with phosphate-buffered saline (PBS; pH 7.2) and lysed in cell lysis buffer (50 mM Tris-HCl containing 2% SDS). Protein contents were measured using a DC protein assay kit (Bio-Rad, Hercules, CA). The samples were subjected to 10% SDS-PAGE, and then transferred to polyvinylidene difluoride membranes (Millipore Corp., Billerica, MA). Nonspecific binding sites were blocked by immersing the membranes in 5% bovine serum albumin in PBS for 1 hour at room temperature, then they were incubated with the primary antibodies; rabbit antidentin sialoprotein (DSP) (Santa Cruz Biotechnology, Inc., Santa Cruz, CA, USA) and rabbit antidentin matrix protein 1 (DMP-1) (Takara Bio, Inc., Shiga, Japan). Subsequently, the membranes were incubated with anti-rabbit and anti-mouse IgG secondary antibodies (GE Healthcare, Little Chalfont, and Buckinghamshire, UK). Immunodetection was performed using an ECL plus Western blot detection system (GE Healthcare), according to the manufacturer's instructions. Blots were stained with Coomassie Brilliant Blue and all lanes were confirmed to contain similar amounts of protein extract.

### 2.6. Statistical Analysis

 Statistical differences were determined using one-way ANOVA computation combined with Scheffe test for multiple comparisons. All data are expressed as the mean ± SD.

## 3. Results

### 3.1. Cell Viability, Morphology, and ALP Activity of KN-3 Cells

**Figure 1:**
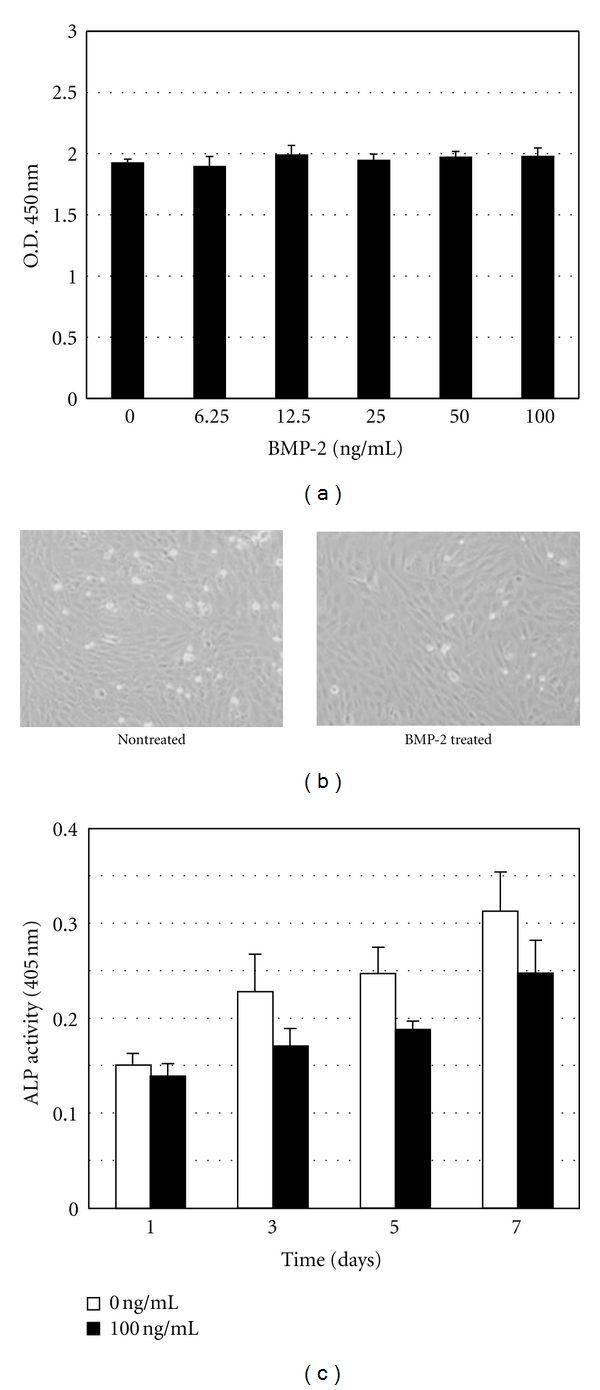
(a) Viability of KN-3 cells after exposure to BMP-2. Data are expressed as the mean ± standard deviation of triplicate cultures. Each experiment was performed 3 times, with similar results obtained in each. (b) Phase-contrast microphotographs of KN-3 cells treated with BMP-2 (100 ng/mL) for 72 hours. (c) Alkaline phosphatase activity of KN-3 cells treated with BMP-2 (100 ng/mL). BMP-2 was replaced with fresh medium every 3 days.

Figure 1 shows the effects of BMP-2 (100 ng/mL) on cell viability, morphology, and ALP activity of KN-3 cells. The results of the WST-1 assay showed no significant differences in cell viability among several different concentrations of BMP-2 (Figure 1(a)). Also, there were no differences in the morphology of KN-3 cells before and after confluence between the presence and absence of BMP-2 (Figure 1(b)). The ALP activity of KN-3 cells after confluence increased in a time-dependent manner, regardless of exposure to BMP-2 (Figure 1(c)).

### 3.2. Intracellular Responses of KN-3 Cells

**Figure 2:**
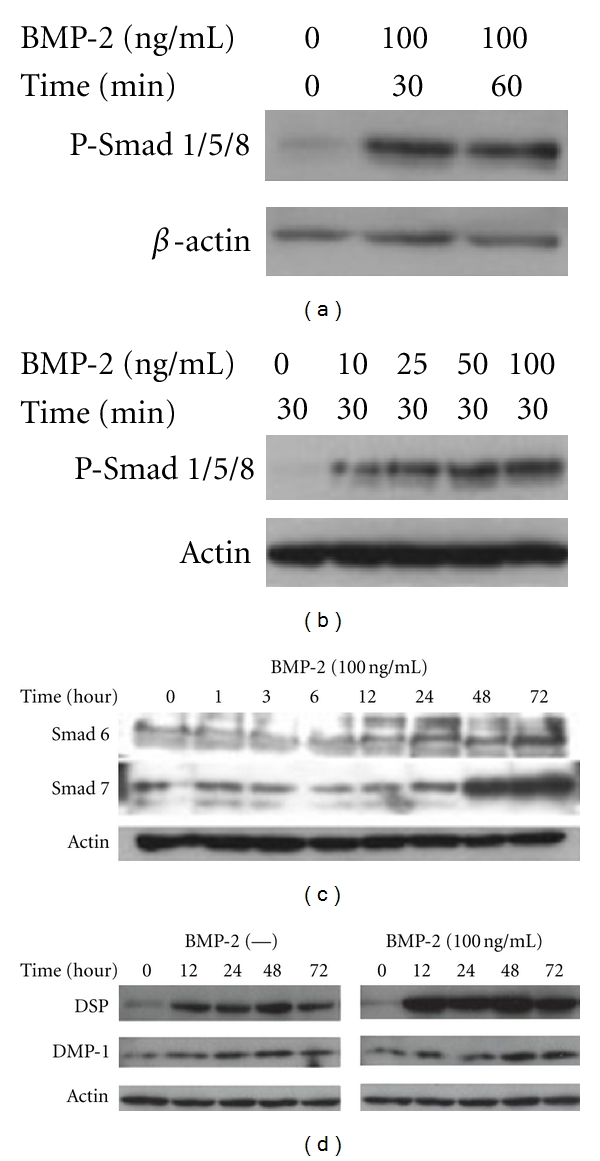
Western blot analysis of (a) and (b) phosphorylated Smad1/5/8, (c) inhibitory Smad6/7, and (d) DSP and DMP-1 extracted from KN-3 cells after treatment with BMP-2.

Figure 2 shows the effects of BMP-2 on the activation of Smad signaling pathways. The addition of BMP-2 (100 ng/mL) induced phosphorylation of Smad1/5/8 in KN-3 cells, which reached a maximum level within 30 minutes (Figure 2(a)). Also, the BMP-2-induced phosphorylation of Smad1/5/8 increased in a dose-dependent manner, and the maximum in the phosphorylation was at 100 ng/mL of BMP-2 (Figure 2(b)). The expressions of Smad6 and 7 appeared to increase at 24 hours and reached a maximum level within 72 hours after the addition of BMP-2 (100 ng/mL) and then continued throughout the culture period (Figure 2(c)).

The expression of DSP and DMP-1, markers of odontoblast differentiation, were increased at 12 and 48 hours, respectively, following the addition of BMP-2 (100 ng/mL), the expression of DSP were increased throughout the culture period, but that of DMP-1 had been on the increase, but not significantly (Figure 2(d)).

## 4. Discussion

A number of studies have used primary dental pulp cells, existing pulp cell lines, and a few odontoblastic cells lines, and characterized their properties to elucidate tooth development, responses of dental pulp to external stimuli, as well as therapeutic approaches for the dentin-pulp complex. Previously, we established KN-3 cells from dental pulp and found that the cells had odontoblastic properties. In the present study, we examined the effects of BMP-2, the most bioactive molecules to induce osteoblast and odontoblast differentiation, on KN-3 cells *in vitro* in order to clarify further characteristics of KN-3 cells as odontoblastic-like cells. BMP-2 had no effects on cell growth, morphology, and ALP activity of KN-3 cells. Previously, we reported that KN-3 cells are able to form calcified nodules. It was also suggested that the outgrowth of odontoblastic process was induced by FGF-2 to a much greater degree than BMP-2, and that base membrane components such as laminin also had effects on the outgrowth of odontoblastic process [20, 21]. Furthermore, it was shown that MC3T3-E1 cells, osteoblastic cell line, differentiated into odontoblasts without upregulation of ALP activity [22], suggesting that an increase in ALP activity is not necessary for odontoblast differentiation. The present results support the notion that BMP-2 is not necessary for morphological change or activation of ALP in the process of differentiation of odontoblast-like cells.

In contrast, the present results revealed that BMP-2 induced activation of intracellular signaling pathways in KN-3 cells, as we found phosphorylation of Smad1/5/8, and up-regulation of Smad6 and 7 in BMP-2-treated KN-3 cells. It is well known that the members of TGF-*β* including BMPs activate Smad proteins are intracellular signaling molecules, and that the Smad pathway is one of important signaling pathways in signal interpretation from the ECM during osteogenesis/dentinogenesis. There are 3 types of Smads: receptor regulated (R-Smads), common mediator (Co-Smads), and inhibitory (I-Smads) [23]. Following the phosphorylation R-Smads (Smad1, 5, and 8) by BMPs, heteromeric complexes are formed with Co-Smad (Smad4) and translocate to the nucleus where they regulate the transcription of target genes together with other nuclear cofactors. In the present study, we clearly detected that BMP-2-induced phosphorylation of Smad1/5/8 in KN-3 cells, as well as upregulation of Smad6 and 7. I-Smads (Smad6 and 7) provide feedback inhibition of BMP-receptor activation by blocking continued R-Smad phosphorylation by BMP receptors, suggesting that upregulation of I-Smads may be essential to limit BMP signaling for proper odontoblastic differentiation of KN-3 cells. Taken together, the present results are the first to demonstrate that KN-3 cells respond to BMP-2 signaling via activation of Smad signaling pathways.

We also examined whether BMP-2 has effects on the expressions of odontoblast specific molecules in KN-3 cells and found that those of DSP and DMP-1 were induced by BMP-2 in a time-dependent manner. DSP is a noncollagenous dentin matrix protein that is transcripted from the dentin sialophosphoprotein (DSPP) gene and known as an odontoblast-specific protein [24–27]. The previous study revealed that KN-3 cells cultured for 72 hours showed high expression level of DSPP [19], indicating that KN-3 cells are precursor cells, which have abilities to differentiate into odontoblast-like cells. DSP increase in BMP-2-untreated cells in the present study was resulted from the properties of KN-3 cells, and the enhanced expression of DSP on BMP-2-treated KN-3 cells indicates the upregulation of odontoblastic differentiation. DMP-1 is a noncollagenous protein expressed in mineralized tissues [24–26, 28]. Various lines of evidence indicate that DSP is induced in the early stage of odontoblast differentiation, whereas DMP-1 is induced in the late stage and tightly bound to the mineral phase of dentin. In the present study, effects of BMP-2 on the early stage of KN-3 cell differentiation were analyzed, resulting in no significant difference in DMP-1 expression between BMP-2-treated and nontreated cells and the less expression of DMP-1 than that of DSP in BMP-2-treated cells.

**Figure 3:**
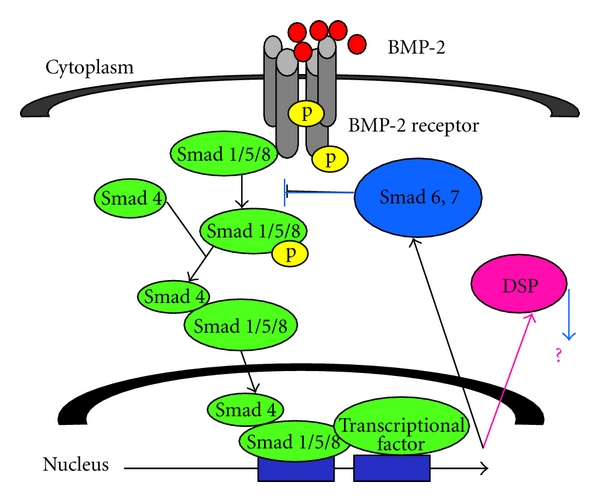
Proposed schema of Smad signaling pathways in KN-3 cells.

 Our results indicate that BMP-2 plays critical roles in induction of the odontoblast properties of KN-3 cells via activation of Smad signaling pathways. In previous studies [29], it was indicated that BMP-2 mediates DSPP expression and odontoblast differentiation and Smad signaling pathway plays a crucial part in the regulation of Dspp expression through the action of Smads, Dlx5, Runx2, and Msx2, suggesting that the expressions of DSP and DMP1 in KN-3 cells may be linked to the Smad pathway (Figure 3). We are continuing research to clarify the direct regulation of DSP and DMP-1, as well as mineralized nodule formation in the later stage of KN-3 cells differentiation, by BMP-2-Smad signaling pathway. Our results also show that KN-3 cells with odontoblastic properties are useful to clarify molecular mechanisms of odontoblasts against external stimuli such as growth factors, in order to develop appropriate regeneration therapy for the dentin-pulp complex.
